# The evolutionary host switches of *Polychromophilus*: a multi-gene phylogeny of the bat malaria genus suggests a second invasion of mammals by a haemosporidian parasite

**DOI:** 10.1186/1475-2875-11-53

**Published:** 2012-02-22

**Authors:** Fardo Witsenburg, Nicolas Salamin, Philippe Christe

**Affiliations:** 1 Département d'Ecologie et Evolution, Université de Lausanne, Biophore, UNIL-Sorge, 1015 Lausanne, Switzerland; 2 Swiss Institute of Bioinformatics, Quartier Sorge, 1015 Lausanne, Switzerland

**Keywords:** *Polychromophilus*, Malaria, Haemosporida, Chiroptera, *Plasmodium*, Host switch, Phylogenetic analysis, Outgroup selection

## Abstract

**Background:**

The majority of Haemosporida species infect birds or reptiles, but many important genera, including *Plasmodium*, infect mammals. Dipteran vectors shared by avian, reptilian and mammalian Haemosporida, suggest multiple invasions of Mammalia during haemosporidian evolution; yet, phylogenetic analyses have detected only a single invasion event. Until now, several important mammal-infecting genera have been absent in these analyses. This study focuses on the evolutionary origin of *Polychromophilus*, a unique malaria genus that only infects bats (Microchiroptera) and is transmitted by bat flies (Nycteribiidae).

**Methods:**

Two species of *Polychromophilus *were obtained from wild bats caught in Switzerland. These were molecularly characterized using four genes (*asl, clpc, coI, cytb*) from the three different genomes (nucleus, apicoplast, mitochondrion). These data were then combined with data of 60 taxa of Haemosporida available in GenBank. Bayesian inference, maximum likelihood and a range of rooting methods were used to test specific hypotheses concerning the phylogenetic relationships between *Polychromophilus *and the other haemosporidian genera.

**Results:**

The *Polychromophilus melanipherus *and *Polychromophilus murinus *samples show genetically distinct patterns and group according to species. The Bayesian tree topology suggests that the monophyletic clade of *Polychromophilus *falls within the avian/saurian clade of *Plasmodium *and directed hypothesis testing confirms the *Plasmodium *origin.

**Conclusion:**

*Polychromophilus*' ancestor was most likely a bird- or reptile-infecting *Plasmodium *before it switched to bats. The invasion of mammals as hosts has, therefore, not been a unique event in the evolutionary history of Haemosporida, despite the suspected costs of adapting to a new host. This was, moreover, accompanied by a switch in dipteran host.

## Background

Five genera belonging to the order of Haemosporida (Apicomplexa) are known to infect mammals: *Plasmodium, Hepatocystis, Polychromophilus, Nycteria *and *Rayella *[1,2]. The dipteran vectors of the first three haemosporidian genera are represented by Culicidae (*Anopheles *spp.), Ceratopogonidae and Nycteribiidae respectively, while the vectors of *Nycteria *and *Rayella *are unknown [1,2]. Culicidae and Ceratopogonidae also act as vectors of the avian and saurian Haemosporida [1,3]. These shared vectors suggest that haemosporidian parasites might have invaded mammals multiple times during their evolution. On the other hand, the switch to mammals is thought to have been an evolutionary demanding process for the parasite [4] and therefore a rare event [5].

Molecular phylogenetic studies to date have been able to detect only a single host switching event to mammals: mammalian *Plasmodium *and *Hepatocystis*, the main mammal-infecting genera, had a common origin and formed a monophyletic sister clade to sauropsid *Plasmodium *[6,7]. However, these phylogenetic studies suffer from incomplete taxon sampling with most investigations including, besides the genera *Plasmodium *and *Hepatocystis*, only the avian *Haemoproteus *and *Leucocytozoon*. Consequently, with no knowledge of the evolutionary origin of the other mammalian haemosporidian groups (i.e. *Rayella, Nycteria, Polychromophilus*), a second move into mammals cannot be excluded.

One possible approach for resolving this standing question is to select a haemosporidian genus that could potentially have switched to mammal hosts independently of mammalian *Plasmodium*/*Hepatocystis*. A good candidate genus for this is *Polychromophilus *as it is well described, with the majority of its life cycle well documented, including its vector stage. Moreover, it infects mammals but is not transmitted by Culicidae like *Plasmodium*, nor Ceratopogonidae like *Hepatocystis*, but by Nycteribiidae (Diptera: Hippoboscoidea). Furthermore, *Polychromophilus' *vertebrate host species range is restricted to the insectivorous bats (Microchiroptera). A phylogenetic analysis of *Polychromophilus *can, therefore, elucidate whether it arose through an independent switch to mammal hosts [8].

**Figure 1:**
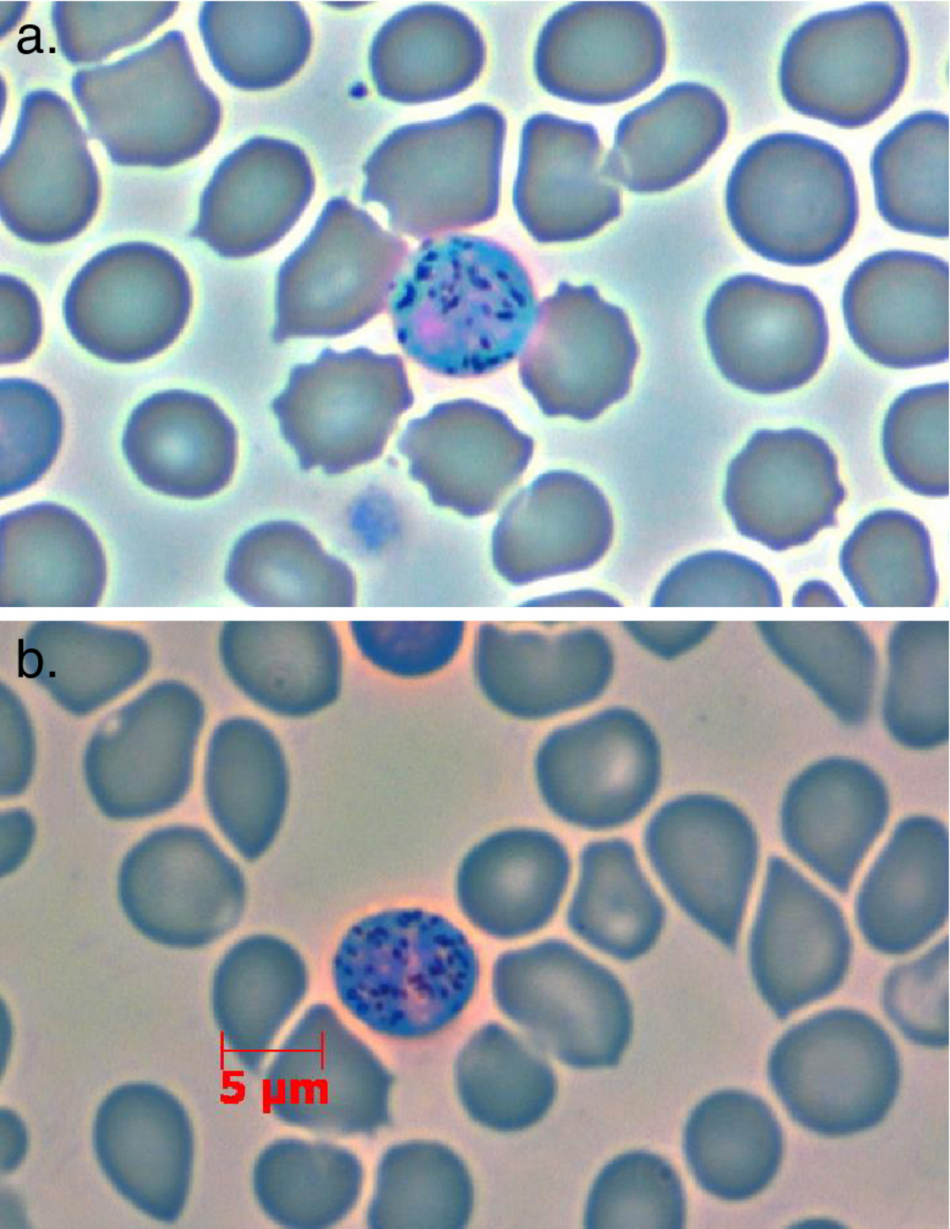
***Polychromophilus murinus *and *P. melanipherus *gametocytes**. a. *Polychromophilus murinus *infection isolated from blood of a Daubenton's bat; b. *P. melanipherus *infection isolated from blood of a Schreibers' bent-winged bat. (Thin blood film, Giemsa-staining, phase contrast image).

Only five species of *Polychromophilus *are known to exist. While they can be distinguished by their slight differences in ultrastructure, they are mainly classified based on host-type [9,10]. Landau et al. [10] proposed dividing the genus into two subgenera based on their gametocyte morphology: 1) the subgenus *Polychromophilus*, with *P. (P.) melanipherus *as the type species, which has gametocytes similar to the type 'malariae'; 2) the subgenus *Bioccala *with type species *Polychromophilus (B.) murinus *whose gametocytes resemble the benign tertian parasites of birds and reptiles (see Figure 1) [10]. Later, it was even suggested that the subgenus *Bioccala *be raised to genus level [11]; however, this was not reflected in the literature [12]. Moreover, the morphological distinctions between the species have been described as 'slight' [9] and how well they reflect the genetics of the genus has not been studied.

The Nycteribiidae vectors are also known as nycteribids or bat flies. These haematophagous flies are completely adapted to a parasitic lifestyle in the fur of bats in that they have lost their wings, have no or reduced eyes and possess hooking claws which allow them swift movements through the fur [13,14]. Coradetti [15] was the first to detect sporozoites in their salivary glands and later studies confirmed his finding [12,16].

**Figure 2:**
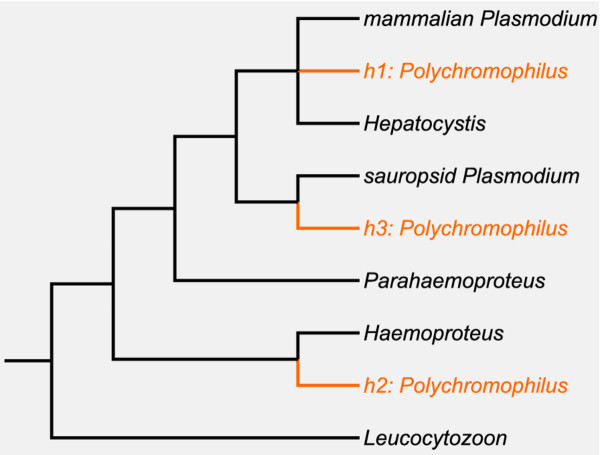
**The hypothetical phylogeny of the genus *Polychromophilus *and the other Haemosporida**. The hypothetical branches are marked in orange and based either on the conservation of the vertebrate host (hypothesis 1), the conservation of the dipteran vector (hypothesis 2), or based on previous molecular studies of the *cytb *gene (hypothesis 3).

When an evolutionary conservation of hosts is assumed, *Polychromophilus*' unique host-vector combination of Mammalia and Nycteribiidae gives rise to two hypotheses on its phylogenetic relationships: 1) it is monophyletic with the mammalian *Plasmodium*/*Hepatocystis *clade with which it shares the vertebrate host type, or 2) it shares its most recent common ancestor with the subgenus *Haemoproteus *(*Haemoproteus*), which has a similar vector. The genus *Haemoproteus *contains two avian subgenera which have different vectors. *H. (Parahaemoproteus) *spp. use biting midges as vectors, and *H. (Haemoproteus) *spp. are transmitted by Hippoboscidae, whose closest relatives are the bat flies [17]. A phylogeny based on ultrastructure and life-history traits grouped *Polychromophilus *together with both subgenera of *Haemoproteus *[8]. However, two recent molecular phylogenetic studies based on part of the cytochrome b sequence both suggest, despite their different topologies, a close relationship between *Polychromophilus *and sauropsid *Plasmodium *[18,19]. This fact provides a third hypothesis: 3) *Polychromophilus *is monophyletic with sauropsid *Plasmodium *(see Figure 2).

The aim of this study was to test these three hypotheses against each other. Though previous studies on the phylogenetic relationships of *Polychromophilus *have been done, all used only a single gene. Different genes in a single organism can show different evolutionary patterns and it is, therefore, recommended to use multiple genes for accurate relationship estimation [20]. The four genes from three different genomes sequenced for this study represent two species of *Polychromophilus *(being the two type species of the two proposed subgenera). These sequences were subsequently combined with an existing dataset of 60 species of Haemosporida to clarify the phylogenetic relationships and gain insight into the evolutionary host switches of *Polychromophilus*.

## Methods

### Sample collection and preparation

Four *Miniopterus schreibersii *(Schreibers' bent-winged bat) were caught using mist nets in the entrance of an abandoned mine in western Switzerland under authorization 2203 issued by the Veterinarian Service of Canton Vaud, Switzerland. Blood was obtained by puncturing the uropatagial vein with a 0.5 mm gauge needle (Neolus). The blood beads that consequently formed on the uropatagium (between 10 and 30 μl total) were taken up in a microvette with EDTA (Sarstedt) and stored at 4°C until further analysis. Haemostatic cotton was applied on the punctured vein until the bleeding had stopped before releasing any bats.

One drop of blood was applied to a glass microscope slide for later visual identification of the parasite species. After smearing the blood, the slide was dried and immediately submerged in 100% methanol for fixation. Finally 5% Giemsa-staining was applied for one hour to stain the cells. DNA was extracted from whole blood using the DNeasy Blood and Tissue kit (Qiagen). Megali et al. [18] provided extracted DNA samples from blood of *Myotis daubentoni *(Daubenton's bat) which contained *P. murinus *infections. These infections were previously shown to be characterized by different cytochrome b haplotypes [18].

### Molecular analysis

For the phylogenetic reconstruction, four genes were selected from the three cellular genomes: two mitochondrial DNA sequences, cytochrome b (*cytb*, 607 bp) and cytochrome oxidase subunit I (*coI*, 768 bp); one DNA sequence from the apicoplast, caseinolytic protease C (*clpc*, 502 bp); and one nuclear DNA sequence, adenylosuccinate lyase (*asl*, 186 bp).

All primer pairs used for the polymerase chain reactions were taken from Martinsen et al. [6] with the exception of *coI nested Po*, which was designed during this study (see Table 1 for primer sequences). All reactions started with an initial denaturation phase at 94°C for four minutes and one minute for the first and nested PCRs, respectively. The reactions ended with an annealing phase at 72°C for 7 min. All cycles started for 30 s at 94°C, but the other cycle conditions and the number of cycles differed depending on the primer pair used (see Table 1). The 25 μl reaction volume contained 3 μl of extraction product, 0.25 U Taq polymerase, 0.3 mM of both primers, 0.25 mM dNTP's, 1× Qiagen PCR buffer and a total of 2 mM MgCl (except for the reactions with the *coI *primers, which had a total of 3 mM MgCl). The nested PCR reaction volume was similar except for the extraction product, which was replaced with 1 μl of product of the first PCR. For the *asl *amplification the first PCR product was purified, which resulted in a better performance of the nested reaction.

**Table 1:** Name, sequence and PCR conditions of the primers used

*Name*		*Primer sequence*	*Annealing*	*Extension*	*Cycles*
asl/outer	fw	GSKAARTTTAATGGKGCTGTWGG	47°C, 30 s	72°C, 50 s	35

	rv	GGATTAAYTTTATGAGGCATTG			

asl/nested	fw	GCTGATMAAAATRTTGATTGG	50°C, 30 s	72°C, 30 s	38

	rv	GAGGCATTGTACTACTWCC			

clpc/outer	fw	AAACTGAATTAGCAAAAATATTA	50°C, 30 s	72°C, 50 s	38

	rv	CGWGCWCCATATAAAGGAT			

clpc/nested	fw	GATTTGATATGAGTGAATATATGG	48°C, 30 s	72°C, 30 s	40

	rv	CCATATAAAGGATTATAWG			

coI/outer	fw	CTATTTATGGTTTTCATTTTTATTTGGTA	57°C, 30 s	72°C, 50 s	35

	rv	AGGAATACGTCTAGGCATTACATTAAATCC			

coI/nested Po	fw	AGCAATATCAATAGCTGCATTACCT	62°C, 30 s	72°C, 50 s	38

	rv	GATTTTCTTCAATATAATGCCTGGA			

cytb/outer	fw	TAATGCCTAGACGTATTCCTGATTATCCAG	55°C, 30 s	72°C, 50 s	35

	rv	TGTTTGCTTGGGAGCTGTAATCATAATGTG			

cytb/nested	fw	TCAACAATGACTTTATTTGG	55°C, 30 s	72°C, 50 s	40

	rv	TGCTGTATCATACCCTAAAG			

All denaturation and final extension periods are the same for all primer-pairs.

All successfully amplified samples were purified according to the manufacturer's protocol using the Wizard PCR Clean-Up system (Promega) or the Minelute PCR Purification kit (Qiagen) in the case of *asl*. DNA concentrations were estimated by visualization on a 1.5% agarose gel with a 100 bp reference ladder (Roche). For the sequencing reactions ~20 ng of purified PCR product, 2 μl Big Dye Terminator v3.1 and 1 μl of 10 mM primer were mixed to a 10 μl volume. Sequence analysis was performed on an ABI Prism 3,100 genetic analyser (Applied Biosystems). Sequence chromatographs were checked for ambiguities with Chromas Lite v2.01 (Technelysium).

### Phylogenetic reconstruction

The obtained sequence data were combined with the same gene sequences of 60 other haemosporidian species obtained from GenBank (see Additional file 1). These 60 species represent the major clades of the Haemosporida, i.e. *Leucocytozoon, Haemoproteus *(*Haemoproteus*), *Haemoproteus *(*Parahaemoproteus*), *Hepatocystis *and *Plasmodium *(including mammalian, avian and saurian). Sequences were aligned with ClustalW as implemented in MEGA version 5 [21]. The single-gene alignments were concatenated using FASconCAT [22].

All phylogenetic reconstructions were done using both Maximum Likelihood (ML) analysis and Bayesian inference (BI). For ML analysis, the PhyML software [23] was used for the single-gene alignments. Since PhyML does not allow for partitioning of the data RAxML [24] was used for the concatenated alignment. Models of nucleotide substitution were GTR + Γ + I for *cytb, co1 *and *clpc *and GTR + Γ for *asl*, as determined by MrAIC [25]. For each analysis, the transition rates of the GTR model, the shape of the Γ-distribution and the proportion of invariable sites were estimated by the program. Both the RAxML and PhyML analyses were assessed by performing 1,000 bootstrap replicates.

For the Bayesian analysis, the same models of character evolution as described for the ML analyses were implemented with MrBayes 3.1.2 [26]. In the concatenated analysis the data were again partitioned by gene, where each partition had its corresponding model and independent parameter estimations. The MCMC algorithm was done with four chains and was run for 20,000,000 generations, sampling every 1,000 generations. Two independent runs were performed to assess convergence to the correct posterior distribution. All parameters were checked for convergence using Tracer v1.5 and the first 10% of samples of each run was discarded as burn-in. All computations were performed on the Vital-IT cluster of the Swiss Institute of Bioinformatics.

### Rooting the tree

Which outgroup to use has been a matter of debate lately. Perkins and Schall [7] identified *Leucocytozoon *as the most primitive clade of the order, using *Theileria *as an outgroup in their analysis of *cytb *sequences. But a recent study by Outlaw and Ricklefs [19] demonstrated that, when using a relaxed molecular clock, *Leucocytozoon *becomes the most derived group, effectively turning the tree inside-out. The authors argue that most ancient divergence should be between the mammal-infecting *Plasmodium *and *Hepatocystis *on the one side, and avian/saurian *Plasmodium*, both subgenera of *Haemoproteus *and *Leucocytozoon *on the other.

For the phylogenetic tree reconstructions, the *Leucocytozoon *spp. were initially selected as the outgroup, but these results were tested for their robustness by redoing the analyses using different rooting methods: 1) forcing the mammalian *Plasmodium*/*Hepatocystis *clade as outgroup instead of *Leucocytozoon*; 2) adding amino acid sequences of the more distantly related *Babesia *spp. as the outgroup (see Additional file 1) and repeating the ML analyses; 3) using the molecular clock methods similar to Outlaw and Ricklefs [19] but with varying priors: a Yule or birth-death tree prior, a strict, a log-normal relaxed or an exponential relaxed clock with a GTR + Γ + I substitution model, 20 million generations sampling every 2,000 generations and two independent MCMC runs using BEAST [27-29].

### Topological tests

The obtained Bayesian majority rule consensus tree was compared with each of the four Bayesian single-gene majority rule consensus trees to rule out any conflict in topology. The Kishino-Hasegawa tests [30] were performed in Treefinder [31]. The tests proved non-significant for all genes but *asl *(see Table 2). This gene was, therefore, removed from the concatenated alignment and a new phylogenetic reconstruction was performed on the remaining genes only.

**Table 2:** The Kishino-Hasegawa topological test results

	*Single gene tree*	*Concatenated 4 genes tree*	
	*lnL*	*lnL*	*pKH*
*asl*	-3561.406	-3642.361	< 0.001*
*clpc*	-7718.852	-7728.157	0.2696
*coI*	-10062.16	-10074.33	0.2336
*cytb*	-6856.617	-6859.401	0.4323

For each gene the likelihood of the phylogeny of that gene was compared to the phylogenetic reconstruction based on all four genes. The log-likelihood values and p-values are shown per gene alignment. Only the asl alignment gives a significantly worse likelihood value for the tree based on the combined data, which indicates conflicting topologies.

For each of the three hypotheses on the *Polychromophilus *origin a corresponding topology was constructed. This was done by restricting the placing of *Polychromophilus *during tree reconstruction in RAxML, forcing it either with the mammal-infecting *Plasmodium*/*Hepatocystis *clade (hypothesis 1), the *Haemoproteus (Haemoproteus) *clade (hypothesis 2) or with the sauropsid *Plasmodium *clade (hypothesis 3). These restricted topologies were then tested together with the topology produced by the maximum likelihood analysis using a Shimodaira-Hasegawa test [32] as implemented in PAML 4 [33].

## Results

The stained slides showed erythrocytes infected with slightly oval-shaped gametocytes (see Figure 1b). The granular appearance and pinkish staining at the nucleus fit the description of *Polychromophilus melanipherus *as given by Garnham [1]. The morphology of the observed gametocytes could therefore be linked to the molecular sequences obtained from the infections (Table 3).

**Table 3:** The haplotypes and corresponding accession numbers for GenBank per sequenced sample per gene

	*asl*		*clpc*		*coI*		*cytb*	
*ind*.	*ht*.	*acc. nb*.	*ht*.	*acc. nb*.	*ht*.	*acc. nb*.	*ht*.	*acc. nb*.
104	Pmu1	JN990725	Pmu1	JN990723	Pmu1	JN990718	Pmu1	JN990712
114	Pmu1	..	Pmu2	JN990724	-	-	Pmu1	..
156	Pmu1	..	Pmu1	..	Pmu2	JN990719	Pmu2	JN990713
A2111	-		Pme3	JN990720	Pme3	JN990714	Pme3	JN990708
A2112	Pme2	JN990726	Pme4	JN990721	Pme4	JN990715	Pme4	JN990709
A2113	-	-	Pme5	JN990722	Pme5	JN990716	Pme5	JN990710
A2114	-	-	-	-	Pme6	JN990717	Pme6	JN990711

Samples 104, 114 and 156 are *Polychromophilus murinus*, sampled from *Myotis daubentoni* and shared some haplotypes. The samples A2111-A2114 are *Polychromophilus melanipherus* from *Miniopterus schreibersii* and never shared haplotypes. For each unique haplotype, the GenBank accession number is mentioned only once in the table. '..': accession number already mentioned. '-': sequencing was unsuccessful.

None of the topologies obtained by independent analyses of the separate genes conflicted with the topology resulting from the concatenated alignment (Kishino-Hasegawa tests: *cytb*: Δlnl = 2.8, p_KH _= 0.432, *coI*: Δlnl = 12.1, p_KH _= 0.234, *clpc*: Δlnl = 9.3, p_KH _= 0.270), except for *asl *(Δlnl = 81.0, p_KH _< 0.001). Despite this strong rejection, both the ML and BI trees of *asl *had only few supported nodes and only closely related pairs were recovered (data not shown). A possible cause of the incongruence detected could be positive selection events in the evolution of the *asl *nuclear sequence [34]. However, analyses performed with Codeml [33] did not show signs of positive selection on the nuclear gene.

Although the reasons for this DNA region to be rejected by the topology tests are unclear, the length of the *asl *gene fragment sequenced in this study is very small (186 bp). This could suggest that random errors are responsible for creating the incongruences observed with this gene. Adding other, and especially longer, nuclear genes would certainly bring more information to test if the evolutionary relationships estimated from the different genomes are congruent or if specific gene trees best represent the evolution of each DNA regions. Different cellular genomes often have different evolutionary histories; even within a single genome not all genes show the same phylogenetic relationships [20].

**Figure 3:**
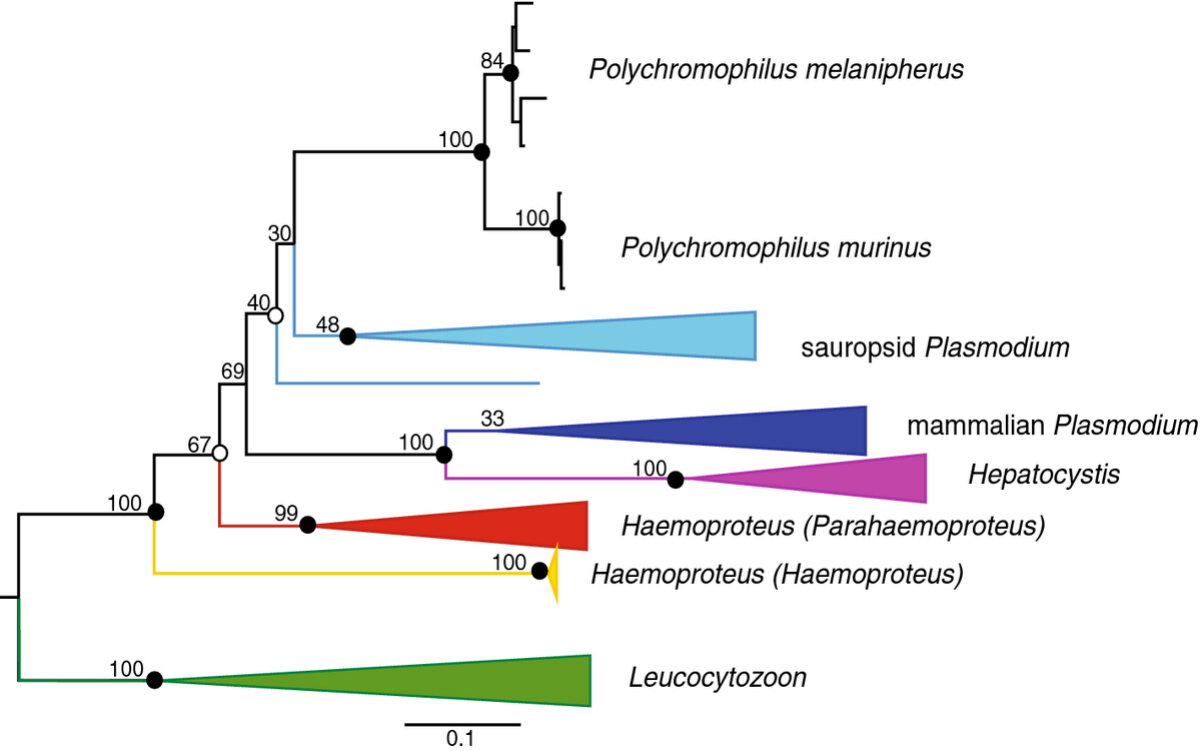
***Polychromophilus *shares its most recent common ancestor with avian and reptilian *Plasmodium***. Shown is the 50% majority-rule consensus tree from the Bayesian inference analysis. The phylogenetic reconstruction using maximum likelihood produced a similar tree. For clarity all clades except the *Polychromophilus *are collapsed and replaced by coloured triangles. Each colour represents a different haemosporidian group. The dots indicate Bayesian node support. Closed dots indicate a posterior probability ≥ 0.95, open dots a posterior probability ≥ 0.90. Node values indicate bootstrap values. Branch lengths represent the number of substitutions. The single blue branch belongs to a *Plasmodium *sp. infecting the skink *Egernia stokesii*.

Figure 3 presents the reconstructed phylogenetic trees using the combined data of *cytb, coI *and *clpc *by ML and BI. The analyses produce no conflict on any of the major nodes. All major genera and subgenera are recovered and represented in the phylogenetic tree by separate monophyletic clades, with the exception of the sauropsid *Plasmodium *clade, which contains *Polychromophilus *within it.

### Diversity of *Polychromophilus *species

*Polychromophilus *forms its own clearly defined clade in both the BI and ML reconstructions. Within this clade, the two species of *Polychromophilus *form well supported separate sister clades (see Figure 3). The distinction between *P. melanipherus *and *P. murinus *has often been made based on host species, since *P. melanipherus *was by definition confined to *Miniopterus schreibersii *as hosts. This distinction, however, has been qualified as 'arbitrary' and 'unsatisfactory' [1]. This study demonstrates for the first time that there is a clear genetic distinction between the two *Polychromophilus *species, confirming their taxonomic status of different species from a molecular point of view.

However, to determine whether this level of genetic divergence between *P. murinus *and *P. melanipherus *merits their placement in different subgenera [10] or even different genera [11], other species of the genus should be added (e.g. *P. deanei *[35] and *P. adami *[10]). Without these supplementary species, the overall observed genetic diversity within the genus *Polychromophilus *is low; it is clearly less than that of the genera *Plasmodium *and *Haemoproteus *or even less than the diversity found in subgenera like *P. (Vinckeia) *and *H. (Parahaemoproteus)*. No critical level of genetic diversity exists as a precondition for the elevation of a subgenus, but the low diversity found within *Polychromophilus *does suggest that confirming *P. (Bioccala) *as a separate genus would cause a taxonomic asymmetry within the Haemosporida.

Two more haemosporidian genera infecting bats have been described: *Dionisia *[36] and *Biguetiella *[11]. Both contain only a single species and are described as 'little different' from *Polychromophilus (Polychromophilus) *[36] and as 'a vicariant form of' *Polychromophilus (Bioccala) *[11], respectively. Whether their similarities to *Polychromophilus *spp. are because of convergence or shared ancestry can only be tested by combining the morphological data with molecular methods [37]. A big obstacle in studying these unfamiliar species however is the lack of observations. No other records of *Biguetiella *or *Dionisia *exist. Single descriptions of new parasite species found in a limited number of hosts are a problem encountered more often by parasitologists and can severely hamper classification [37].

### *Polychromophilus' *placement in the phylogeny of Haemosporida

The bootstrap value (69/100) suggests that the *Polychromophilus *clade is restricted to the *Plasmodium *branch of the haemosporidian tree. Even though this node also appears in the Bayesian majority rule consensus tree, the support for it is actually very weak (posterior probability = 0.73). However, the alternative hypothesis 2, that *Polychromophilus *shares its most recent common ancestor with the subgenus *Haemoproteus (Haemoproteus)*, is clearly rejected (Shimodaira-Hasegawa test; see Table 4).

**Table 4:** The Shimodaira-Hasegawa topological test results comparing the three hypothetical topologies

*Tree*	*lnL*	*p_SH_*
Best tree (hypothesis 3)	-25126.753	-
Hypothesis 1	-25130.147	0.578
Hypothesis 2	-25154.740	0.023*

The best tree was the tree provided by the maximum likelihood analysis (see Figure 3) and concurred with hypothesis 3. The log-likelihoods of the other two trees, based on hypothesis 1 and 2 (see Figure 2), are compared with the best tree. The hypothesis 2 tree, which has *Polychromophilus* grouped with *Haemoproteus*, has a significantly worse fit and can be rejected.

It is less clear where within the *Plasmodium *clade *Polychromophilus *belongs. Neither phylogenetic method indicates that *Polychromophilus *originated from mammalian *Plasmodium*/*Hepatocystis *and both instead produced topologies suggesting a sauropsid origin (see Figure 3). However, the actual support for the node separating the mammalian clade from sauropsid *Plasmodium/Polychromophilus *clade is low. The BI supports the monophyly of sauropsid *Plasmodium *and *Polychromophilus *(hypothesis 3) with a posterior probability of 0.92, but the ML support of that same critical node is absent (bootstrap value of 40/100). The topological test comparing the different phylogenetic scenarios did not provide more support for either hypothesis 1 or 3 (see Table 4).

Most of the alternative rooting methods favour hypothesis 3. Indeed, rooting the tree with the mammalian *Plasmodium *clade instead of *Leucocytozoon*, as suggested by Outlaw and Ricklefs [19], validates the conclusion of a sauropsid origin of *Polychromophilus *in both BI and ML (see Additional file 2).

The choice of the prior distributions guiding either the distribution of mutation rates across the tree (log-normal vs exponential) or the divergence times (Yule vs birth-death) does not change the conclusion. All the molecular clock analyses place *Polychromophilus *within the sauropsid *Plasmodium *clade, with clade credibilities between 0.87 and 1. However the root itself does change depending on the prior set. The Yule and log-normal prior lead to the placement of the *Leucocytozoon *as the outgroup, whereas the mammalian *Plasmodium*/*Hepatocystis *clade is placed as the outgroup with the birth-death tree and exponential relaxed clock (see Additional file 2).

The ML analysis using *Babesia *as an outgroup produces a topology with very little support. All major nodes have bootstrap values of < 50/100, so no outgroup can be identified, nor can *Polychromophilus *be placed within the tree with any confidence (see Additional file 3). The genetic divergence between *Babesia *(Piroplasmida) and the Haemosporida is very high, which results in a very long branch leading to the *Babesia *lineages. This changes the rooting procedure to a problem of 'long-branch attraction' with all corresponding biases [38], and these analyses should therefore be approached with caution [19].

None of the used phylogenetic methods reject our third hypothesis, stating that *Polychromophilus *is monophyletic with the sauropsid *Plasmodium *clade. ML and the topological test could not discriminate between hypothesis 2 and 3, but BI and molecular clock rooting methods gave more support for the latter hypothesis. These analyses are far from conclusive, but do suggest that *Polychromophilus *did not evolve from a mammal-infecting ancestor, but has instead invaded the mammalian class of hosts independently.

Our results show that the three DNA regions used in the combined matrix do not provide sufficient phylogenetic information to unambiguously place the *Polychromophilus *lineage. When combining regions from different genomes, this could introduce sufficient conflict to reduce the confidence in the reconstructed trees, even if the topology tests did not identified major incongruence. The way forward to clearly place the *Polychromophilus *lineage within the large *Plasmodium *clade is to sequence longer stretches of DNA regions, in particular from the nuclear genome, and to use gene tree approaches to identify the best evolutionary relationships at the species level [39,40].

### Previous findings

The close relation between *Polychromophilus *and avian Haemosporida has been suggested before. Carreno et al. [8] produced a phylogeny based on life-history and ultra-structure characters and concluded that *Polychromophilus *is most closely related to *Haemoproteus*, a hypothesis rejected by the current study. Megali et al. [18] used a 705 bp *cytb *fragment and concluded that *Polychromophilus *shared its closest common ancestry with avian *Plasmodium*. However, the base of their tree was not well resolved. The authors themselves recommended the use of multiple genes.

Duval et al. [41] discussed bat Haemosporida, but never identified the species. However, their molecular analyses, again using only *cytb*, grouped their samples clearly with sauropsid *Plasmodium*, leading to a similar conclusion as the current study. In their paper, they cautiously did not name their collected species. However, the corresponding sequences that are available in GenBank have been identified as '*Hepatocystis *sp.'. Based on the work of Megali et al. [18] it is very likely that part of those sequences are actually *Polychromophilus *species. Misidentification is a big obstacle in apicomplexan research as a whole [42] and haemosporidian research in particular [43]. Therefore, caution is required when naming species for GenBank.

### Switch of host, switch of vector

Parasitizing a new, mammalian host likely necessitated many adaptive changes, given their characteristic, non-nucleated red blood cells. The *cytb *DNA region sequenced here showed long branches of non-synonymous substitutions separating the avian from the mammal clade [4]. Many lineages have become extinct over time during the evolution towards the mammalian and avian *Plasmodium *lineages [44]. Nevertheless *Polychromophilus*' origin suggests that the switch to mammalian hosts happened at least twice during Haemosporida evolution. *Rayella *is thought to have originated from *Hepatocystis *[45] and has been classified as such [1], but *Nycteria*'s origins are more elusive; whether it is a case of yet another independent host switch, or an ancient mammalian *Plasmodium *lineage that has survived the pruning on that branch, remains to be investigated.

Haematophagy has appeared multiple times in the evolution of the Diptera. It evolved once at the origin of the superfamily Hippoboscoidea and is shared by all its members [17]. Consequently, many Hippoboscoidea spp. are implicated in the transmission of diseases, most notably sleeping sickness (Glossinidae) and malaria (Hippoboscidae and Nycteribiidae). The relatively high relatedness of the latter two families [17] is not reflected by their haemosporidian parasites. This study convincingly rejected the hypothesis that hippoboscid-transmitted *H. (Haemoproteus) *shares its most recent common ancestor with the nycteribid-transmitted *Polychromophilus*. A cospeciation event of these Haemosporida with their dipteran hosts can, therefore, clearly be excluded.

Instead, *Polychromophilus' *ancestor must have been vectored by of a member of the Culicidae, as are all modern *Plasmodium *species. Culicidae are one of the oldest members of the Diptera, an order with a higher radiation of species than all terrestrial vertebrates put together [46]. The phylogenetic distance between Culicidae and Nycteribiidae is one of the largest within the order [46], yet the adaptations required for this new vector were seemingly acquired in parallel to those required for the new mammalian host.

Because the Nycteribiidae are completely specialised to bats, the first appearance of *Polychromophilus *in bats must have been mediated by either mosquitoes or via the hippoboscid flies. Many Culicidae spp. feed on both mammals and birds readily, and within the Hippoboscidae, the host switch from mammals to birds has happened several times [17]. Therefore, both could have been responsible for the first transmission. However, once *Polychromophilus*' ancestor was introduced in bats, adapting to the nycteribid vectors likely had large fitness advantages. Specifically, the haematophagous lifestyle of both males and females combined with their high prevalence on bats [13], and ease of moving between bat-hosts (unpublished observations), make the Nycteribiidae an ideal vector for the protozoan parasite. However, this same switch to Nycteribiidae also limited the potential range of *Polychromophilus *vertebrate hosts to the Chiroptera.

## Conclusions

The phylogenetic reconstruction of three genes of *Polychromophilus *spp. demonstrates that the *P. melanipherus *and *P. murinus *are clearly two genetically distinct species. Only the addition of the other *Polychromophilus *spp. can validate the current division of *Polychromophilus *in separate subgenera. *Polychromophilus *is clearly not related to *Haemoproteus (Haemoproteus)*. Instead Bayesian inference and molecular clock outgroup free phylogenetic reconstructions suggest that the *Polychromophilus *most likely had a bird- or reptile-infecting *Plasmodium *ancestor. The switch to mammalian hosts would, therefore, not have occurred once, but at least twice in the haemosporidian evolutionary past. This event was accompanied by the adaptation to a new, phylogenetically distant dipteran vector.

## Competing interests

The authors declare that they have no competing interests.

## Authors' contributions

FW collected the samples, carried out the molecular work, performed the phylogenetic analyses and drafted the manuscript. NS participated in the phylogenetic analyses. PC conceived the study, collected the samples and interpreted the results. All authors read and approved the final manuscript.

## Supplementary Material

Additional file 1 **Species name, host and accession numbers of sequences retrieved from GenBank for the phylogenetic reconstructions**. This table contains additional host information and the GenBank accession numbers of all genes used for the phylogenetic analyses. Not all gene sequences are available for all species, missing sequences are denoted by '-'. Click here for file

Additional file 2 **Changing topologies for alternative rooting methods**. Changing topologies acquired by different methods of phylogenetic reconstruction. Irrespective of the root, *Polychromophilus *remains nested within the sauropsid *Plasmodium *clade. a. The original best tree from maximum likelihood reconstruction, but now rooted with the mammalian *Plasmodium*/*Hepatocystis*, as suggested by Outlaw and Ricklefs [16]. Topologies b. and c. are acquired using a relaxed molecular clock with no predefined root, GTR + Γ + I substitution model, 20 million generations sampling every 2,000 generations and two independent MCMC runs using BEAST. All nodes have clade credibilities > 0.5 b. Topology acquired with the Yule tree prior and an exponential relaxed clock. c. Topology acquired with the birth-death tree prior and a log-normal relaxed clock. The different haemosporidian clades are represented by the coloured triangles. The clade height represents the number of containing taxa. Click here for file

Additional file 3 **A topology rooted with *Babesia *provides little information**. The amino acid alignment provides too little contrast to construct a tree with high support as most nodes are unsupported. A very long branch separates the *Babesia *species from all Haemosporida. Shown is the best tree of a ML analysis using a JTT + Γ + I substitution model and bootstrapping a 1,000 times. Closed dots: bootstrap value > 90; Open dots: bootstrap values > 50. Click here for file
