# Supplementary figures and images for: The evolutionary host switches of Polychromophilus: a multi-gene phylogeny of the bat malaria genus suggests a second invasion of mammals by a haemosporidian parasite
Source: Malar J. 2012 Feb 22;11:53. doi: 10.1186/1475-2875-11-53 (PMC3342143; doi:10.1186/1475-2875-11-53)

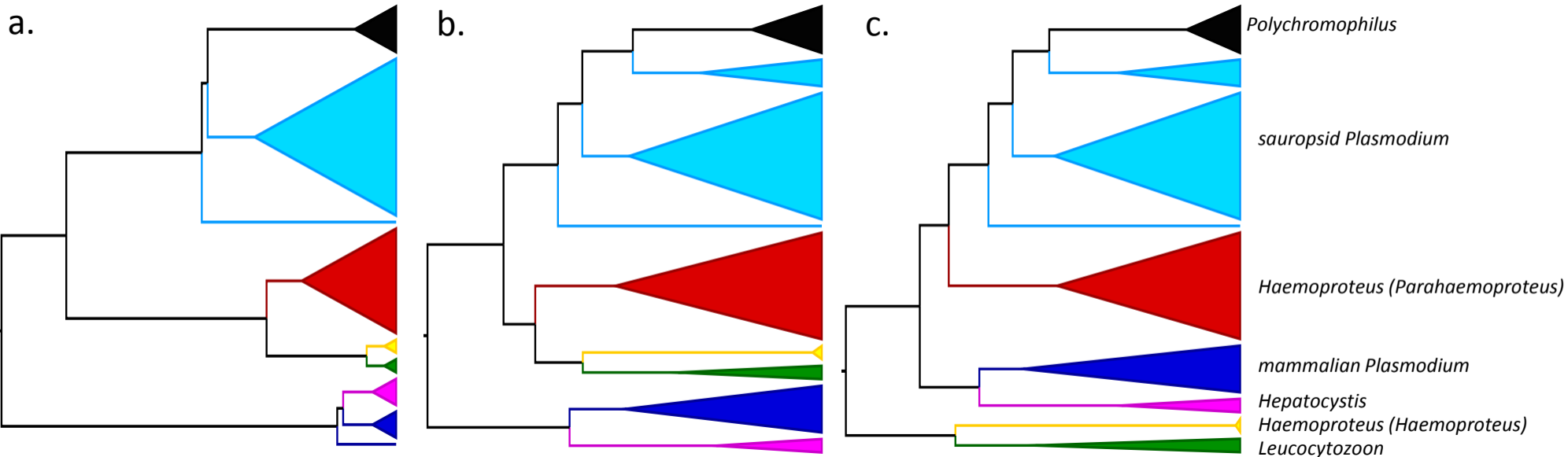

Supplement: Additional file 2 — Changing topologies for alternative rooting methods. Changing topologies acquired by different methods of phylogenetic reconstruction. Irrespective of the root, Polychromophilus remains nested within the sauropsid Plasmodium clade. a. The original best tree from maximum likelihood reconstruction, but now rooted with the mammalian Plasmodium/Hepatocystis, as suggested by Outlaw and Ricklefs [16]. Topologies b. and c. are acquired using a relaxed molecular clock with no predefined root, GTR + Γ + I substitution model, 20 million generations sampling every 2,000 generations and two independent MCMC runs using BEAST. All nodes have clade credibilities > 0.5 b. Topology acquired with the Yule tree prior and an exponential relaxed clock. c. Topology acquired with the birth-death tree prior and a log-normal relaxed clock. The different haemosporidian clades are represented by the coloured triangles. The clade height represents the number of containing taxa. [file 1475-2875-11-53-S2.PDF]

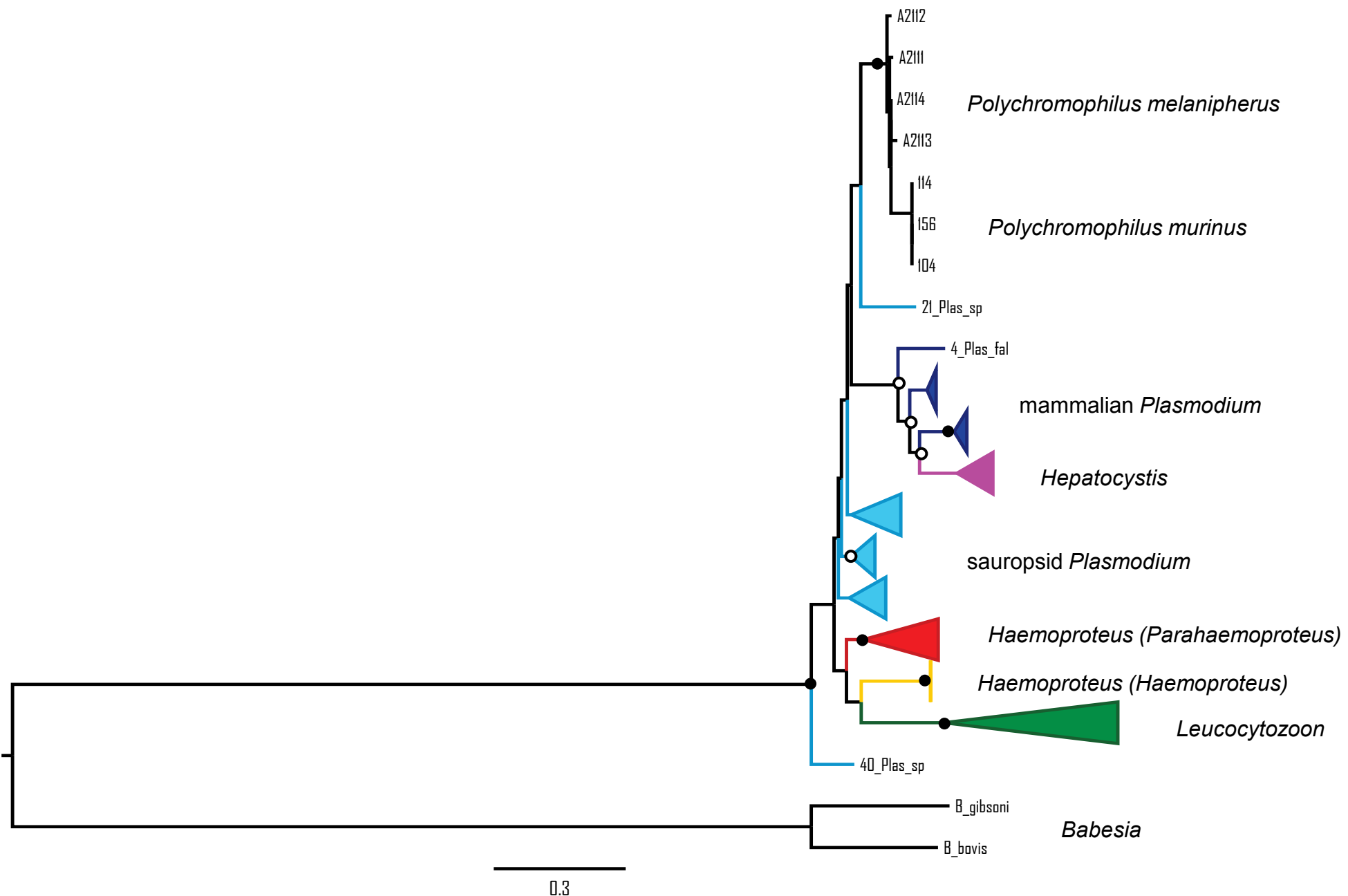

Supplement: Additional file 3 — A topology rooted with Babesia provides little information. The amino acid alignment provides too little contrast to construct a tree with high support as most nodes are unsupported. A very long branch separates the Babesia species from all Haemosporida. Shown is the best tree of a ML analysis using a JTT + Γ + I substitution model and bootstrapping a 1,000 times. Closed dots: bootstrap value > 90; Open dots: bootstrap values > 50. [file 1475-2875-11-53-S3.PDF]
